# Supplementary material for: Discovery and Validation of Methylation Signatures in Circulating Cell-Free DNA for the Detection of Colorectal Cancer
Source: Biomolecules. 2024 Aug 13;14(8):996. doi: 10.3390/biom14080996 (PMC11353097; doi:10.3390/biom14080996)
Supplement: Supplementary file 1 [file biomolecules-14-00996-s001.zip › biomolecules-3040891-supplementary.pdf]

Supplementary Methods

DNA isolation and bisulfite conversion

For tissues and WBCs, genomic DNA was extracted by the standard phenol-chloroform procedure and using the QIAamp DNA Mini Kit (Qiagen). cfDNA was extracted from 3 to 5 mL plasma using the QIAamp Circulating Nucleic Acid Kit (Qiagen). The concentration of cfDNA samples was measured on a Qubit 3.0 Fluorometer using a Qubit™ dsDNA HS Assay Kit (Thermo Fisher Scientific). The extracted DNA samples and control DNAs were bisulfite converted using the EZ DNA Methylation-Gold™ Kit (Zymo Research). All extractions or bisulfite conversion of DNA were performed according to the manufacturer's guidelines.

Whole Genome Bisulfite Sequencing

A total amount of 5.2 microgram genomic DNA spiked with 26 ng lambda DNA were fragmented by sonication to 200-300bp with Covaris S220, followed by end repair and adenylation. Cytosine-methylated barcodes were ligated to sonicated DNA as per manufacturer's instructions. Then these DNA fragments were treated twice with bisulfite using EZ DNA Methylation-Gold Kit (Zymo Research), before the resulting single-strand DNA fragments were PCR amplified using KAPA HiFi HotStart Uracil + ReadyMix (2X). Library concentration was quantified by Qubit 2.0 Fluorometer (Life Technologies, CA, USA) and quantitative PCR, and the insert size was assayed on Agilent Bioanalyzer 2100 system. The library preparations were sequenced on an Illumina Hiseq 4000 platform and 125bp/150bp paired-end reads were generated. Image analysis and base calling were performed with Illumina CASAVA pipeline, and finally 125bp/150bp paired-end reads were generated.

For WGBS data analysis, we first used Fastp software for data quality control and Trimmomatic software (version 0.36) with default parameters for removing adapters and low-quality bases. After quality control and trimming, the reads were aligned using Bismark (version 0.22.3) and the GRCh37 reference genome from UCSC. The next step after alignment is to remove the PCR duplication using deduplicate_bismark. BamUtil (version 1.0.14), was used to examine whether pair-end reads overlapped, and

the overlapped part was trimmed from one end to prevent counting twice from the same observation. By calculating the CT conversion rate of lambda DNA in the process of building the database, the data with a conversion rate of 99% was analyzed later. The total 5mC methylation level was calculated from the number of C and T of all CG loci in the sample population: 5mC-level = C/C + T. We used the R package DSS to identify methylation difference regions (DMRs). DMR was considered to be significant with a fold change > 2 and an adjusted P value < 0.05 (Benjamini–Hochberg). Additionally, we carried out regional annotations on the detected DMRs, connecting the differential expression genes on each DMR with regional information by analyzing factors such as promoter, exon, intron, gene body, up and downstream, ect., including upstream 2 k. ClusterProfiler package in R software was used to investigate Gene Ontology (GO) and Encyclopedia of Genes and Genomes (KEGG) enrichment analysis. $P < 0.05$ was used for the cut-off criterion. GO enrichment histogram of DMR-related genes can visually reflect the number distribution of DMR-related genes in BP (biological process), CC (cell component) and MF (molecular function). KEGG is the main public database on the pathway.

Targeted methylation quantification by quantitative methylation specific PCR

Tissue DNA and cfDNA methylation were measured using quantitative methylation-specific PCR (qMSP). Bisulfite-treated DNA from each specimen served as the template, and qMSP was performed with iTaq Universal SYBR Green Supermix (Bio-Rad, USA) and a Bio-Rad CFX96 Touch (Bio-Rad, USA) while strictly following the operating protocols. Primers were designed using ABI Primer Express software (version 3.0.1) or manually. Primer sequences are listed in Table S2. For the amplification, the total volume of the PCR mixture was 20 μL, including 10 μl of SYBR Green Supermix, 0.6 μl each of forward and reverse primers and their concentrations are both 10 umol/L, 1 μl of bisulfite-converted tissue DNA (5ng/μl) or 7 μl of bisulfite-converted cfDNA, finally add RNase-free water 7.8μl or 1.8μl. The reaction protocol was as follows: 1) Target gene, 6 min at 95°C followed by 42 cycles at 95°C for 30 s, 60°C for 30 s (DAB1)/66°C for 30 s (PPP2R5C, FAM19A5), and 72°C for 30 s; and 2)

housekeeping gene (MyOD), 6 min at 95°C followed by 42 cycles at 95°C for 30 s, 56°C for 30 s, and 72°C for 30 s. All experimental procedures were performed twice to ensure accuracy, and 10% to 20% of the samples were tested a second time to verify the consistency of the results in our study. We prepared the methylation standards by purchasing the Human Methylated & Non-Methylated DNA Set from ZYMO RESEARCH. These standards underwent bisulfite conversion to prepare them for subsequent analysis. In the preparation of the target sequence gradient dilution plasmid sample, the target sequences were purchased from MiaoLing Plasmid Platform, Wuhan, China (http://www.miaolingbio.com/), and confirmed by targeted sequencing. The plasmids were subjected to a series of ten-fold serial dilutions based on their fragment lengths, resulting in copy number gradients of 10^7, 10^6, 10^5, 10^4, 10^3, 10^2, and 10 copies. These diluted plasmids were then used as templates for PCR amplification to establish standard curves. In our study, the purple curve in the figure from left to right is the target sequence gradient dilution plasmid sample, and the red curve is the methylation standard and cancer tissue sample of colorectal cancer patients. According to the fluorescence data obtained during the separation process, the specificity of the amplified products was determined by melting curve analysis. The tissue sample's methylation level was calculated by the following formula: methylation level = (quantity of target gene/quantity of housekeeping gene) × 100%. Here, ' quantity ' refers to the number of copies of the gene measured by qMSP. Regarding cfDNA samples, the methylation-specific PCR is qualitative.

Digital droplet PCR (ddPCR) analysis

The cfDNA methylation analysis was performed on the QX200™ Droplet Digital™ PCR System (Bio-Rad). The total volume of the PCR mixture was 21 μL, containing 10 μL of QX200 ddPCR EvaGreen Supermix (Bio-Rad, USA) , A volume of 0.6 μl each of forward and reverse primers,possessing a concentration of 10umol/L, was utilized, and 7-8 μL template DNA. The heat-sealed plates were placed in a T100 Thermal cycler and amplified for 40 cycles to the endpoint. The PCR conditions were as follows: 5 min at 95 °C (Enzyme activation), 40 cycles of 95 °C for 30s (Denaturation) and 60 °C for 1 min (Annealing/extension); then, 5 min at 4 °C, 5 min at 90 °C (Enzyme deactivation),

4°C hold. The ramp rate was set at 2 °C/s for all steps. Four control samples were included in each ddPCR plate: a methylated DNA control, an unmethylated DNA control, an RNase-free water control, and a nontemplate control (NTC). The methylated DNA control contains DNA with known methylation at the target sites, ensuring that the assay can accurately detect methylation. The unmethylated DNA control contains DNA without methylation at the target sites, confirming that the assay specifically detects methylated DNA. The NTC contains all PCR components except the DNA template and is used to detect contamination in the PCR reagents, while the RNase-free water control uses RNase-free water in place of the DNA template to check for contamination in the water itself. Data from the QX200 Droplet Reader were analyzed in QuantaSoft version 1.7.4 (Bio-Rad). The fluorescence thresholds for calling droplets positive or negative were set manually based on the distribution of droplets of control samples. The same threshold was applied to all the wells of one PCR plate. In the EvaGreen channel, samples with at least two droplets with fluorescent amplitudes above the threshold were considered positive.

Supplementary Results

Comparison of qMSP and ddPCR Detection Methods

Eight samples, including 6 cases and 2 controls, were randomly selected for comparative assessment using both qMSP and ddPCR methods to evaluate DMRs methylation in cfDNA. Results are presented in Figure S7-8. Notably, the qMSP method's melting curves revealed non-specific amplification in cases 2, 5, and 6, whereas the ddPCR method showed positive results for case 2 and negative results for cases 5 and 6. Case 1 remained undetectable by both methods. Cases 3 and 4 were confirmed positive for methylation by both qMSP and ddPCR. In the control group, both control 1 and control 2 yielded negative results with both qMSP and ddPCR methods (Figure S8). In summary, the qMSP system demonstrated sensitivity and specificity comparable to ddPCR in detecting DMRs methylation in cfDNA from CRC patients. The system exhibited a remarkable detection limit as low as 100 copies, effectively distinguishing non-specific amplification.

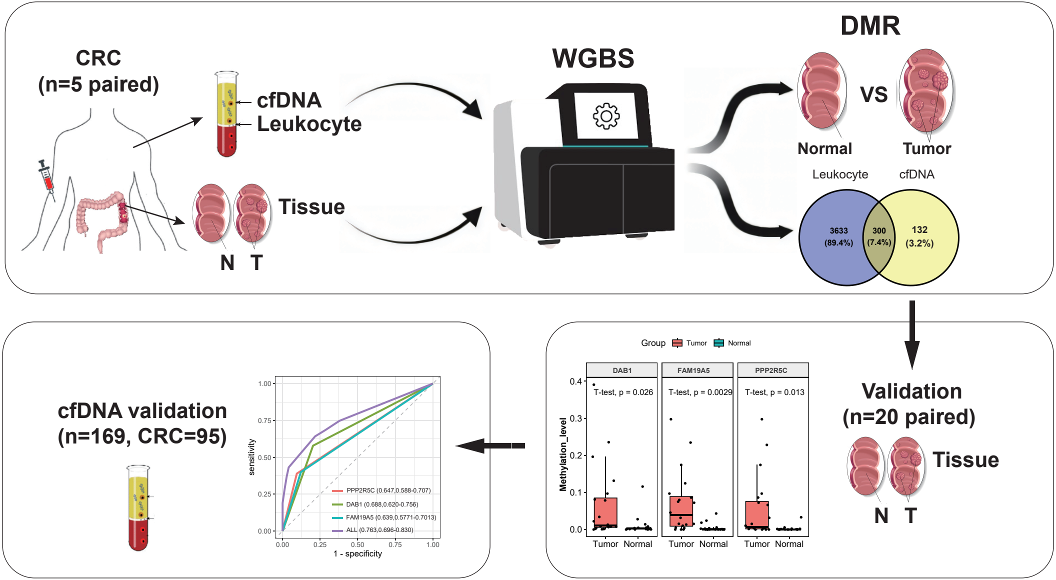

Supplementary Figure 1. The overall structure and methods used for methylation analysis and DMRs screening and validation in colorectal cancer.

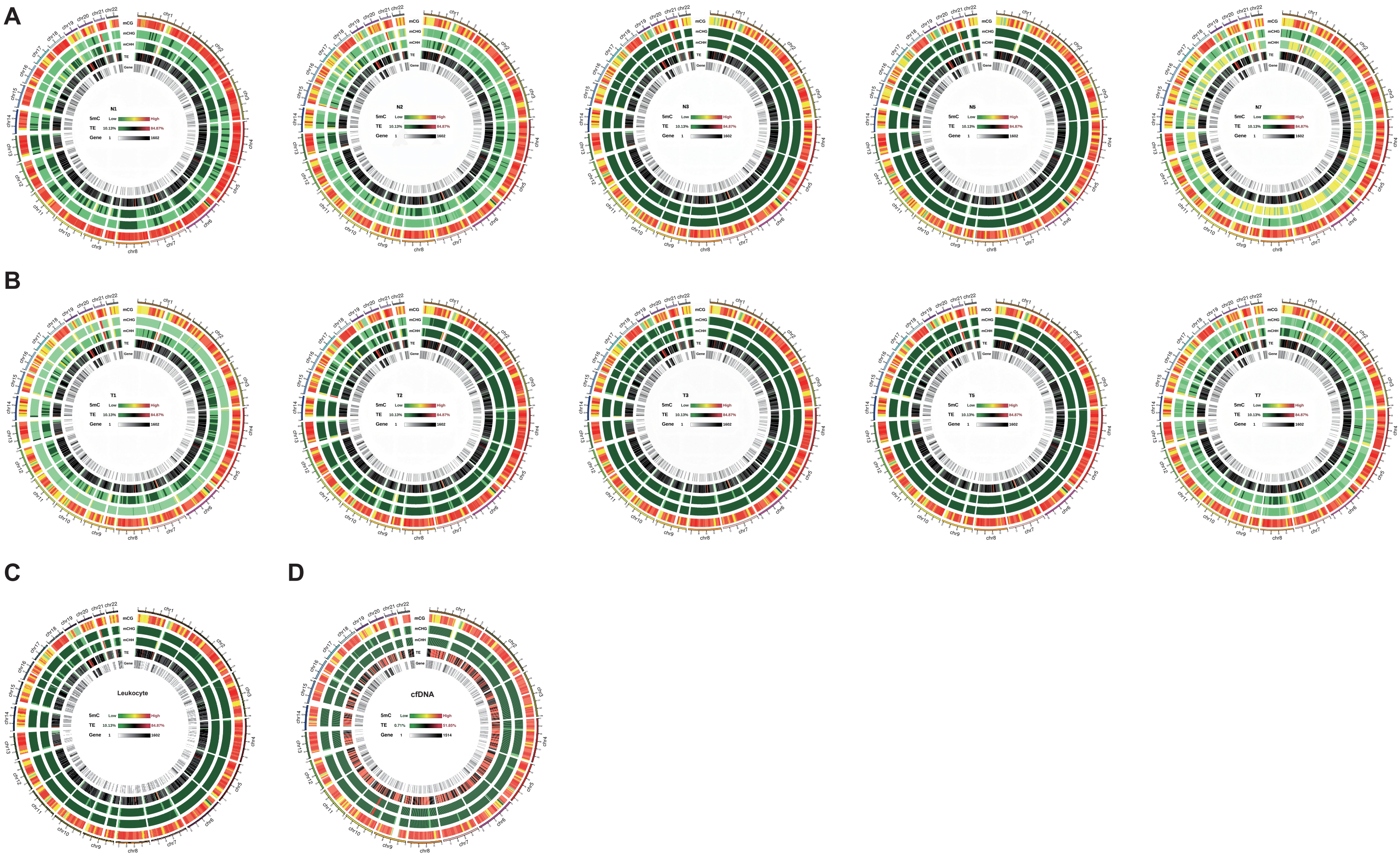

Supplementary Figure 2. Circos map of chromosome methylation density in different samples. (A) adjacent normal tissues. (B) tumor tissues. (C) peripheral blood leukocytes sample. (D) cfDNA sample.   Note: From the outside to the inside, it is shown successively: CG sequence environmental methylation density, CHG sequence environmental methylation density, CHH sequence environmental methylation density, transposable element(TE) component proportion density heat map, gene number density heat map; Inter-nal scale: MC density heat label: from green to yellow to red, methylation density is from low to high; TE ratio heat label: from green to black to red, repeat sequence ratio is from low to high; gene density heat label: from gray to black, gene number is from low to high. TE density calculation method: The TE ratio is calculated as the proportion of repetitive sequence length within a genomic bin, which is a subdivided segment of the genome for analytical purposes. If no repeats are present, the analysis is not performed; gene density calculation: Calculate the number of genes contained in each bin.

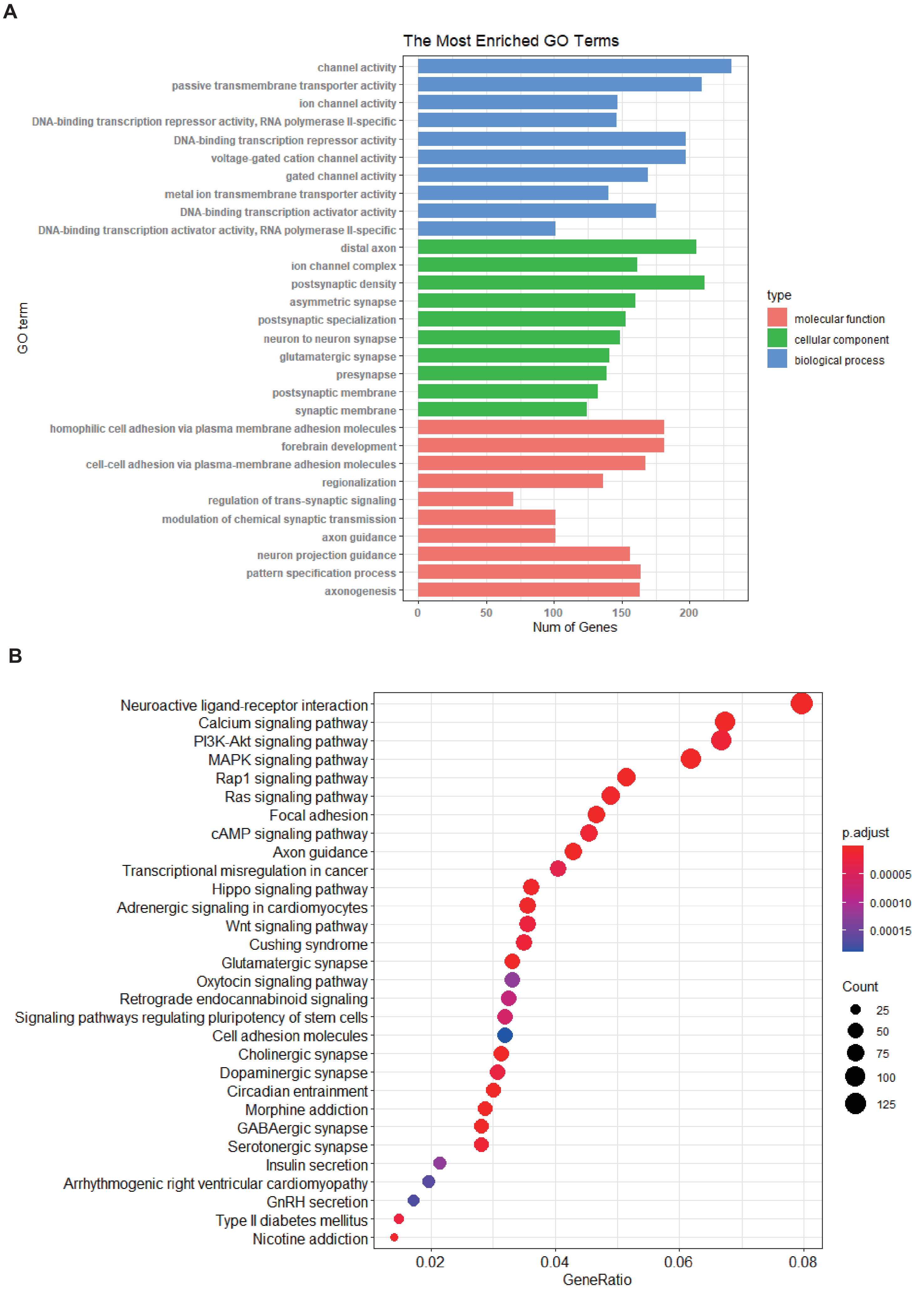

Supplementary Figure 3. The function analysis of DMGs. (A) GO terms enriched analysis of DMGs. (B) Scatter plot of the top 30 KEGG enrichments analysis.

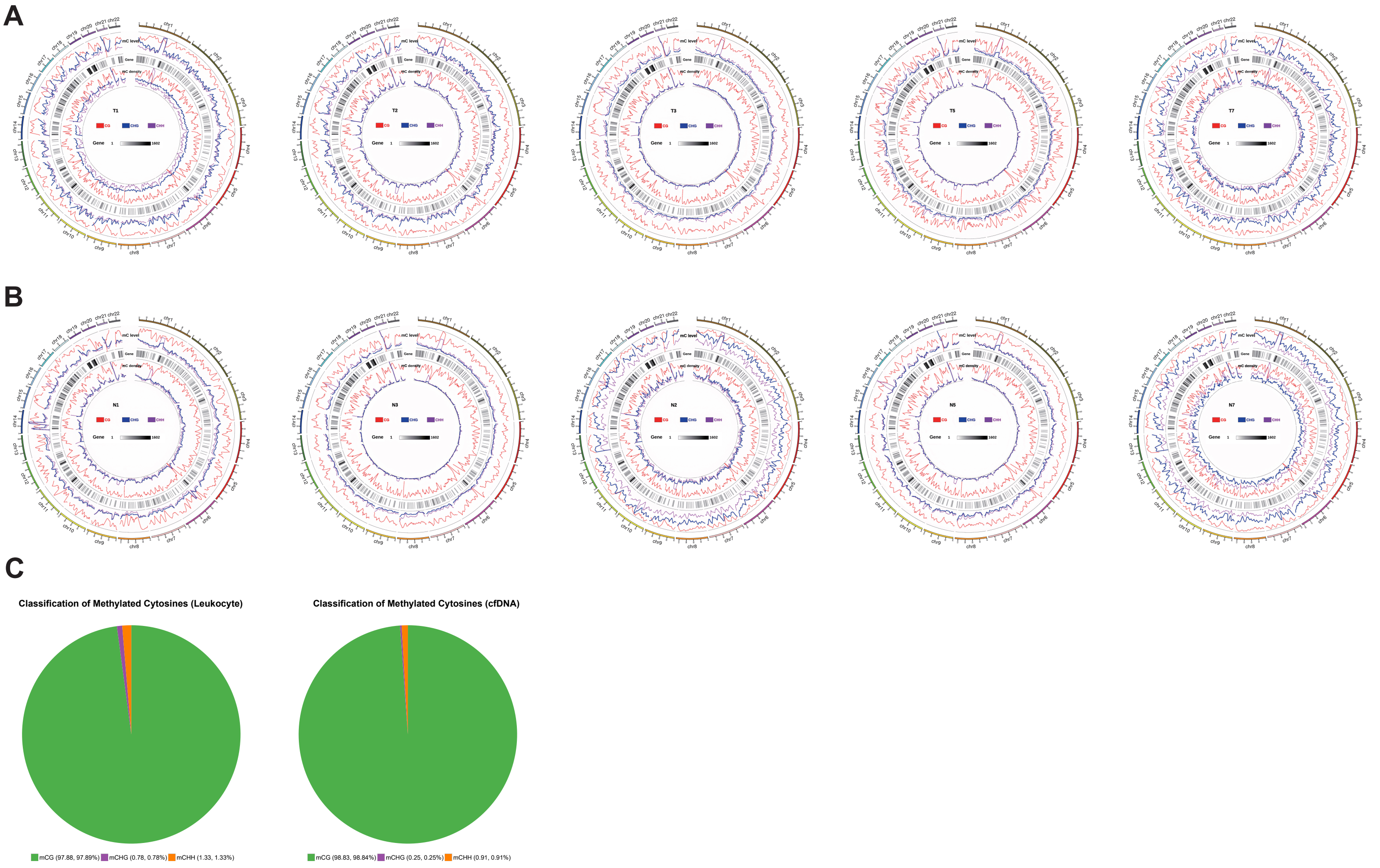

Supplementary Figure 4. Circos map of chromosome methylation level and proportional distribution map of methylation C site. (A) Circos map of chromosome methylation levels in tumor tissue. (B) Circos map of chromosome methylation levels in adjacent tissues. (C) The proportion distribution of methylation C sites in peripheral blood leukocytes and cfDNA samples.

Note: Circos map, from the outside to the inside, methylation level is displayed linearly,gene number density heat map is displayed linearly; Internal scale: three sequence environments (CG in red, CHG in blue; CHH is purple), gene density heat scale: from gray to black indicates the number of genes from low to high.

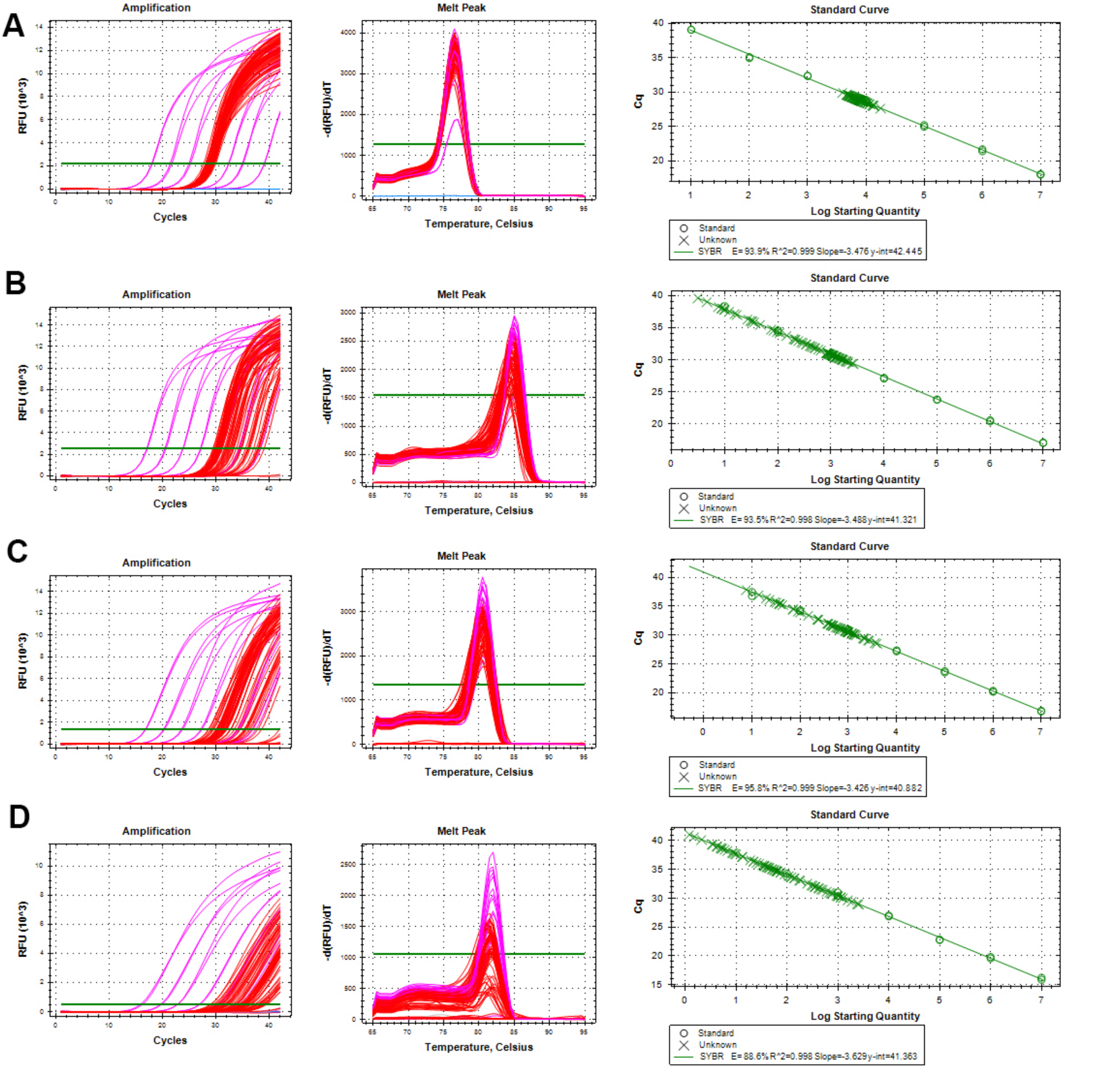

Supplementary Figure 5. Quantitative Methylation-Specific PCR (qMSP) Curves for Methylation Analysis of Genes HK (MyOD) and DMRs in DAB1, PPP2R5C, and FAM19A5 Genes in Colorectal Cancer Tissue DNA.
Note: The purple curve in the figure from left to right is the target sequence gradient dilution plasmid sample, and the red curve shows methylation standards and cancer tissue samples from colorectal cancer patients.
(A) qMSP curve of HK methylation in tissue DNA, from left to right is Amplification curve, Melt peak and Standard curve. The R^2 value was 0.999, and Slope was -3.476. (B) qMSP curve of DAB1 methylation in tissue DNA, from left to right is Amplification curve, Melt peak and Standard curve. The R^2 value was 0.998, and Slope was -3.488. (C) qMSP curve of PPP2R5C methylation in tissue DNA, from left to right is Amplification curve, Melt peak and Standard curve. The R^2 value was 0.999, and Slope was -3.426. (D) qMSP curve of FAM19A5 methylation in tissue DNA, from left to right is Amplification curve, Melt peak and Standard curve. The R^2 value was 0.998, and Slope was -3.629.

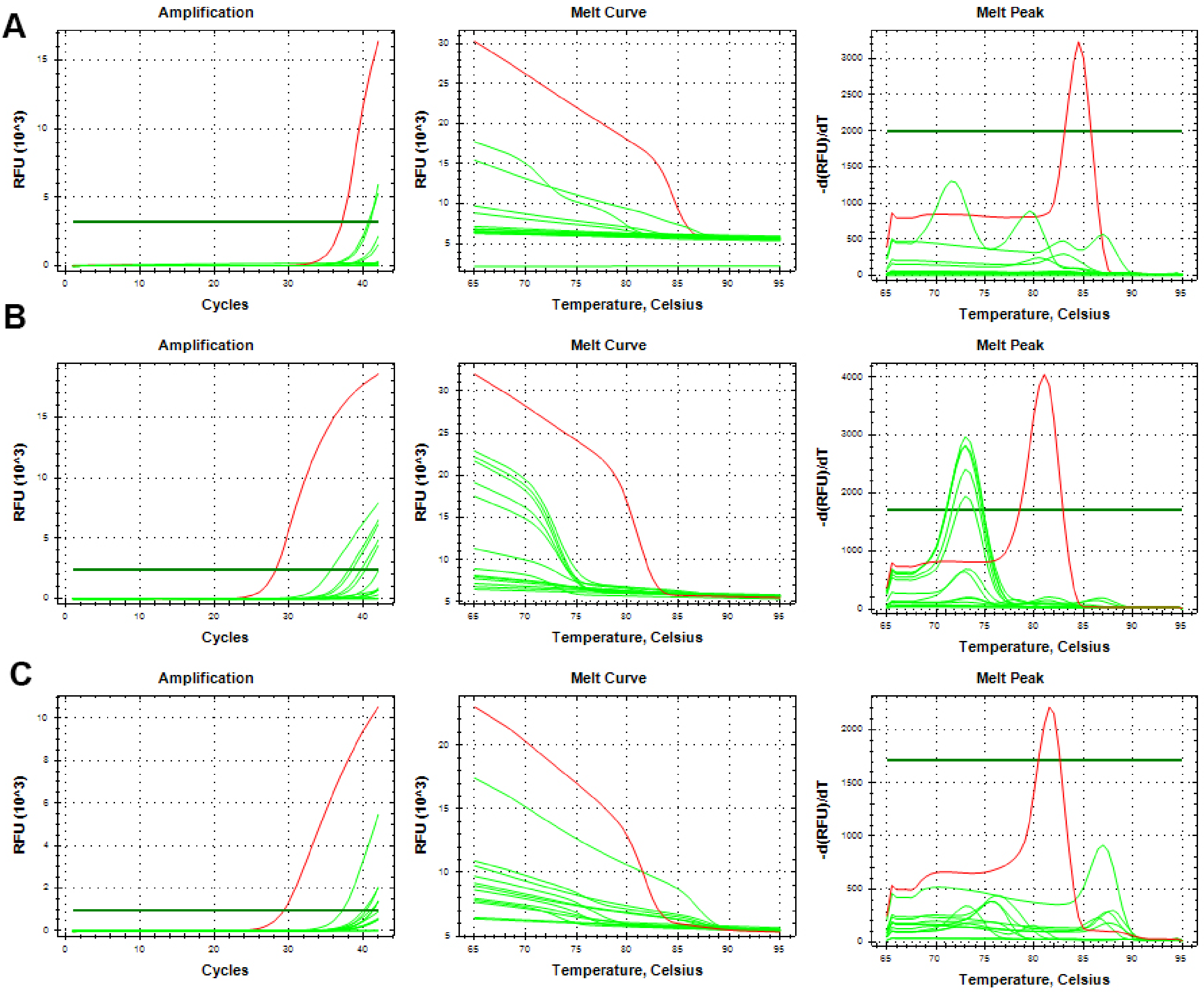

Supplementary Figure 6. Quantitative Methylation-Specific PCR (qMSP) Curves for Methylation Analysis of DMRs in DAB1, PPP2R5C, and FAM19A5 Genes in Colorectal Cancer Leukocyte DNA.

Note: Red is the amplification and melting curve of the standard product, and green is the amplification and melting curve of DAB1, PPP2R5C and FAM19A5 in colorectal cancer leukocyte DNA. (A) qMSP curve of DAB1 methylation in leukocyte DNA, from left to right is Amplification curve, Melt curve and Melt peak. (B) qMSP curve of PPP2R5C methylation in leukocyte DNA, from left to right is Amplification curve, Melt curve and Melt peak. (C) qMSP curve of FAM19A5 methylation in leukocyte DNA, from left to right is Amplification curve, Melt curve and Melt peak.

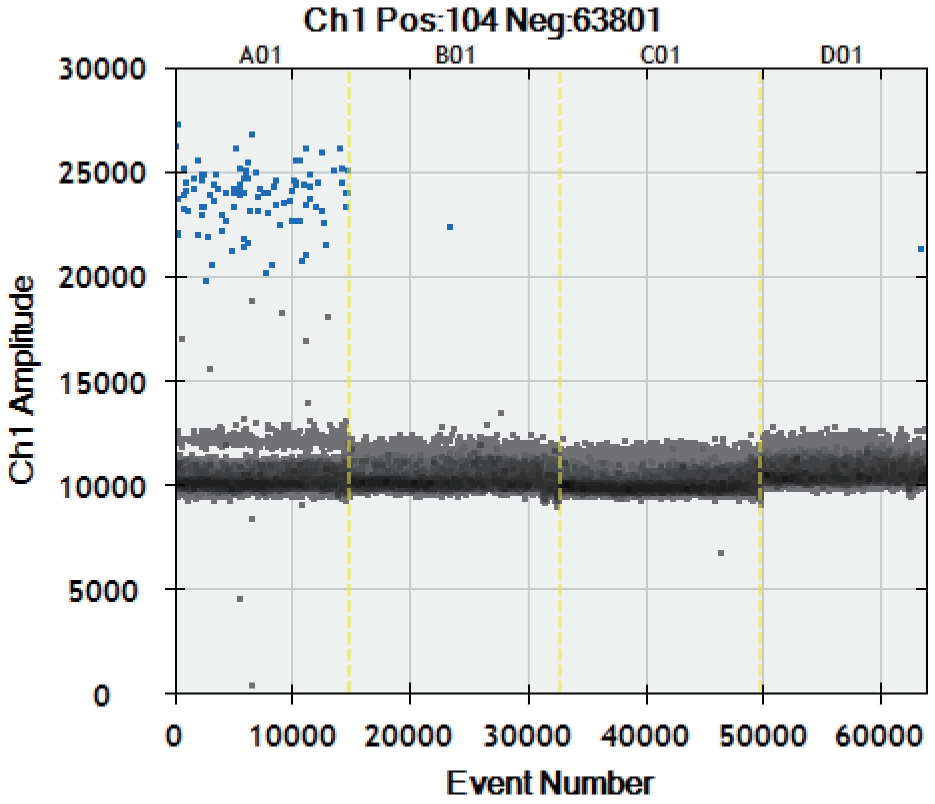

Supplementary Figure 7. Quantasoft Amplification of DMR Methylation in the DAB1 Gene in cfDNA of Colorectal Cancer Determined by ddPCR

Note: Samples from left to right are methylated control, unmethylated control, RNase-free water and non-template control

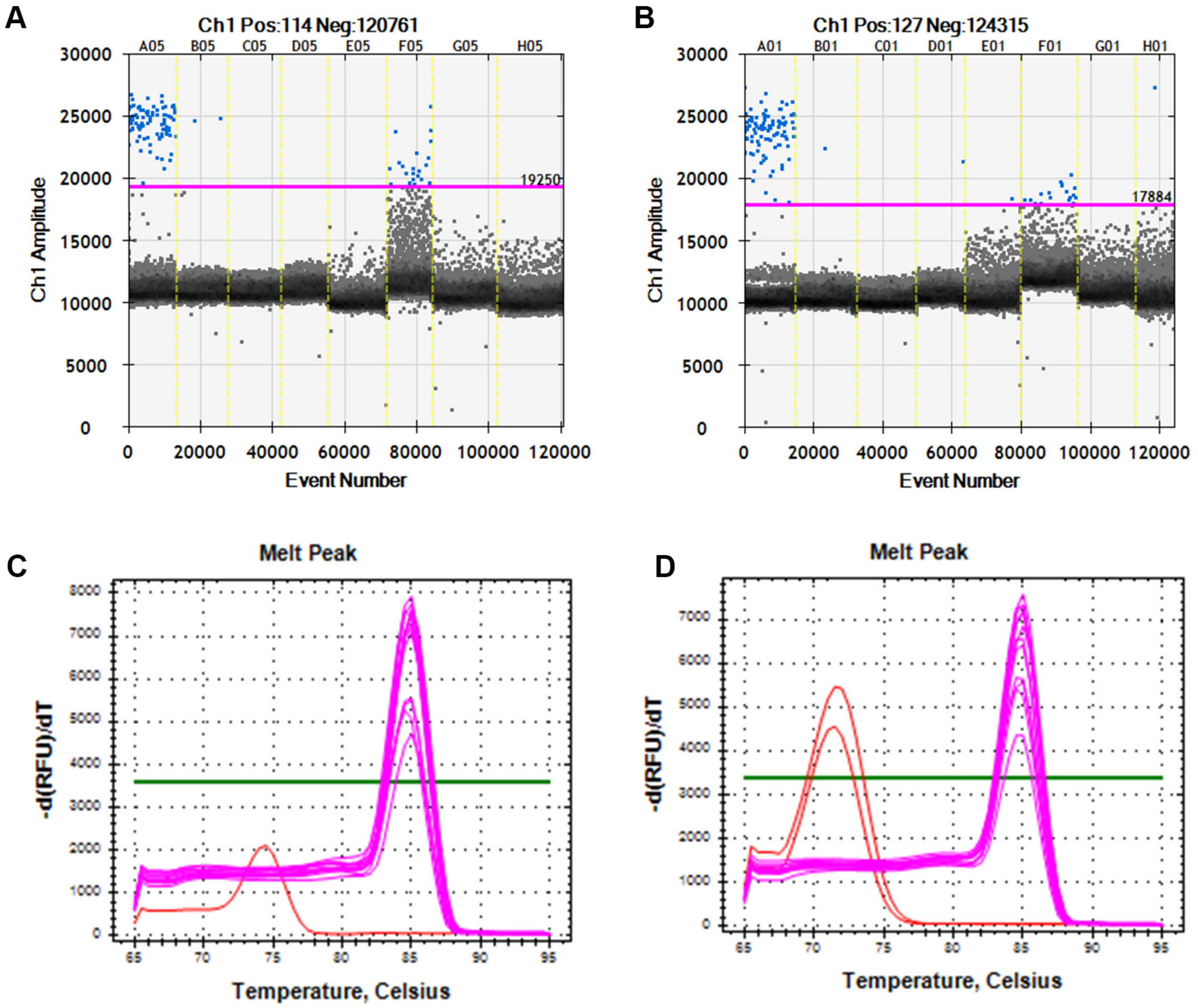

Supplementary Figure 8. Quantasoft Amplification and Melting Curves for DMR Methylation in the DAB1 Gene in cfDNA of Colorectal Cancer Determined by ddPCR and qMSP.

Note: (A) Samples from left to right are methylated control, unmethylated control, RNase-free water and non-template control, case 1, case 2, control 1, control 2. (B) Samples from left to right are methylated control, unmethylated control, RNase-free water and non-template control, case 3, case 4, case 5, case 6. The solid pink lines are manually set thresholds that distinguish between positive and negative droplets. (C) Red is the melting curve of case 2 in qMSP detection, and purple is the melting curve of DMRs plasmid amplification. (D) Red is the melting curve of cases 5 and 6 in qMSP detection, and purple is the melting curve of DMRs plasmid amplification.

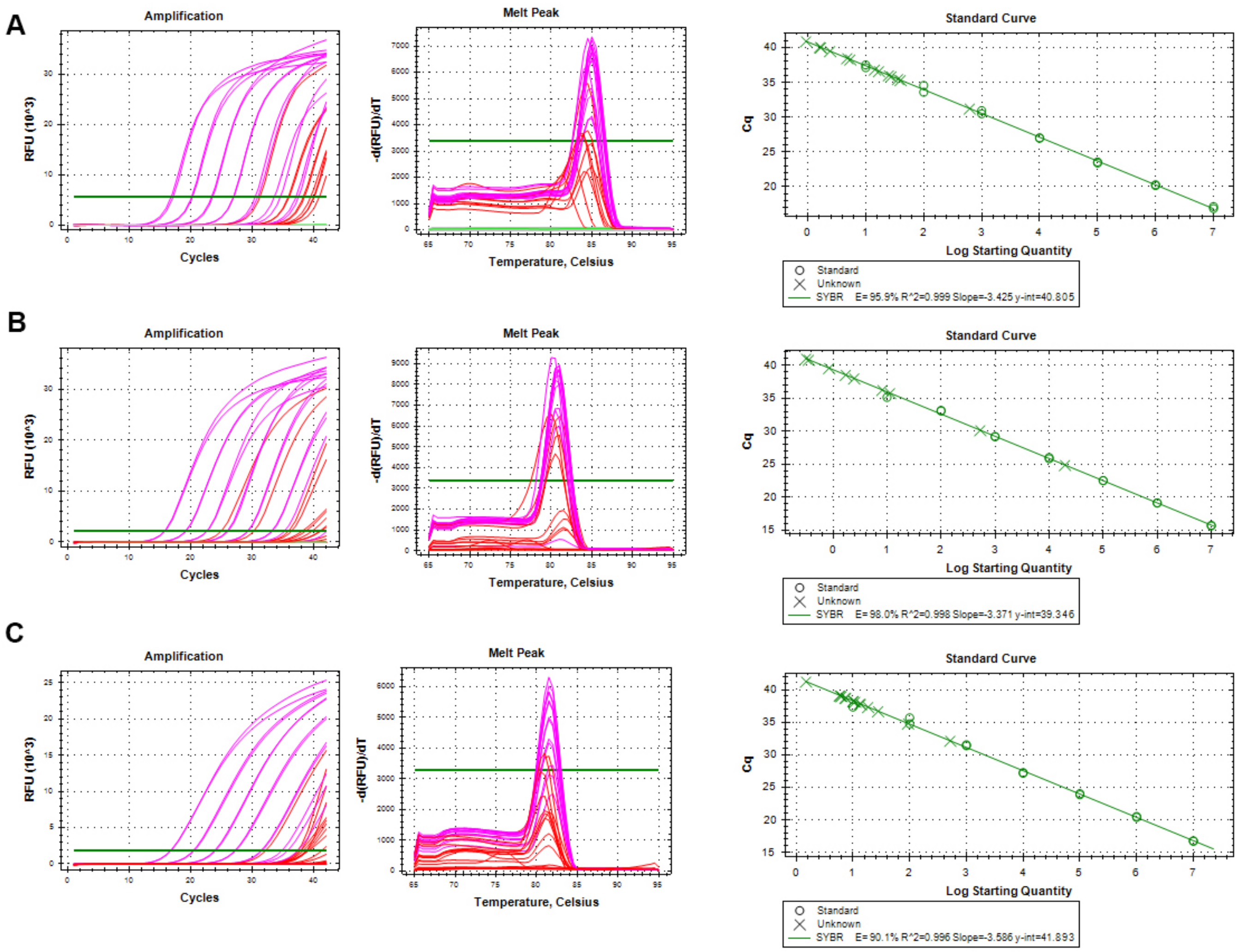

Supplementary Figure 9. Quantitative Methylation-Specific PCR (qMSP) Curves for Methylation Analysis of DMRs in DAB1, PPP2R5C, and FAM19A5 Genes in Colorectal Cancer cfDNA.

Note: Red is the amplification and melting curve of the standard and the measurement sample, and purple is the amplification and melting curve of the standard.

(A) qMSP curve of DAB1 methylation in cfDNA, from left to right is Amplification curve, Melt peak and Standard curve. The R^2 value was 0.999, and Slope was -3.425. (B) qMSP curve of PPP2R5C methylation in cfDNA, from left to right is Amplification curve, Melt peak and Standard curve. The R^2 value was 0.998, and Slope was -3.371. (C) qMSP curve of FAM19A5 methylation in cfDNA, from left to right is Amplification curve, Melt peak and Standard curve. The R^2 value was 0.996, and Slope was -3.586.

Supplementary Table1.1. Demographic Characteristics of Colorectal Cancer Patients in Whole-Genome Bisulfite Sequencing (WGBS) Analysis

Sample ID	Gender	Age	T stage	N stage	M stage
T1/N1	Male	55	T3	N1	M0
T2/N2	Male	59	T3	N0	M0
T3/N3	Female	51	T3	N0	M0
T5/N5	Female	61	T3	N1	M0
T7/N7	Female	71	T2	N0	M0

Note: T, tumor tissue; N, adjacent normal tissues

Supplementary Table 1.2. The demographic characteristics of the CRC and healthy controls

**Characteristics**	**CRC tissues（%）**	**CRC plasma（%）**	**Healthy controls（%）**
Total	20	95	74
Age (years)		*P* value 0.26	
<60	9（45.0）	36（36.0）	35（45.8）
⩾60	11（55.0）	57（64.0）	37（54.2）
Mean ± SD	59.05±12.57	62.53±10.98	60.46 ±12.49
Gender		*P* value 0.003	
Male	12（60.0）	54（56.8）	24（33.3）
Female	8（40.0）	41（43.2）	48（66.7）
UICC stage			
I- II	7（46.7）	42（63.6）	-
III-IV	8（53.3）	24（36.4）	-
T stage			
T1-T2	1（6.7）	14（20.6）	-
T3-T4	14（93.3）	54（79.4）	-
N stage			
N0	9（60.0）	47（69.1）	-
N1/N2	6（40.0）	21（30.9）	-
M stage			
M0	14（93.3）	67（98.5）	-
M1	1（6.7）	1（1.5）	-
Pathological type			
protruded type	1（6.7）	6（9.2）	-
Ulcerative type	14（93.3）	59（90.8）	-
Differentiation			
Poorly differentiated	2（13.3）	10（15.6）	-
Intermediate differentiation	9（60.0）	44（68.8）	-
Well differentiated	4（26.7）	10（15.6）	-
Perineural invasion			
Yes	13（86.7）	47（73.4）	-
No	2（13.3）	17（26.6）	-
Vascular invasion			
Yes	5（33.3）	28（43.1）	-
No	10（66.7）	37（56.9）	-

Supplementary Table 2. Primer sequences of qMSP

Gene	Primer	Primer Sequence (5'-3')	Strand	Genomic Location
HK (MyOD)	Forward	CCAACTCCAAATCCCCTCTCTAT	+	chr11:30009730-30017030
	Reverse	TGATTAATTTAGATTGGGTTTAGAGAAGGA		
DAB1	Forward	ATTTAGTTCGGCGTTCGTTCG	+	chr1:57423822-57424446
	Reverse	CGACGCTAAACTCAAAAATTACCG		
PPP2R5C	Forward	AGTTTTCGTCGGTCGTTTTCG	+	chr14: 101781526-101781974
	Reverse	AACCAAATCGACGTCCTCCG		
FAM19A5	Forward	TTTATTCGGGTTTCGGGCG	+	chr22: 48489799-48490522
	Reverse	GCATCTCGACCGAAAAACCG		

Supplementary Table 3. Location and Methylation Levels of 132 Differentially Methylated Regions Identified by WGBS in Colorectal Cancer and Detected in cfDNA

chr	start	end	length	nCG	T_meanMethy	N_meanMethy	diff.Methy	areaStat	gene_symbol	annotation
chr16	89639215	89639277	63	5	0.955665	0.334843	0.620822	35.12394	DPEP1	Promoter
chr9	1.13E+08	1.13E+08	272	28	0.87979	0.380026	0.499764	210.4982	FAM225B	Exon
chr19	38507118	38507217	100	6	0.651306	0.212146	0.439159	40.25097	RYR1	Intron
chr4	54233	54337	105	7	0.876599	0.534693	0.341905	38.60008	ZNF595	Promoter
chr1	1427575	1427924	350	57	0.363056	0.096251	0.266805	373.8849	TMEM88B	Promoter
chr4	1.9E+08	1.9E+08	422	7	0.835419	0.586994	0.248425	34.63407	DBET	Exon
chr1	31155161	31155300	140	17	0.343485	0.108155	0.23533	83.15284	NKAIN1	Distal Intergenic
chr4	1.9E+08	1.9E+08	214	53	0.442598	0.20959	0.233008	414.5923	FRG2	Exon
chr7	1.54E+08	1.54E+08	479	97	0.403786	0.175806	0.22798	819.7848	DPP6	Promoter
chr13	1.09E+08	1.09E+08	520	114	0.522767	0.298527	0.22424	860.7323	MYO16-AS2	Exon
chr22	45007166	45007728	563	101	0.306021	0.08466	0.22136	702.2822	PHF21B	Promoter
chr12	1.32E+08	1.32E+08	267	60	0.360306	0.140736	0.21957	343.0398	GALNT9	Promoter
chr12	1.32E+08	1.32E+08	220	20	0.695991	0.481165	0.214826	108.3247	NOC4L	Distal Intergenic
chr1	34929254	34929666	413	112	0.301215	0.090583	0.210633	868.8795	DLGAP3	Promoter
chr7	1.03E+08	1.03E+08	1021	5	0.642629	0.433123	0.209507	21.37106	RASA4B	Intron
chr7	1229661	1229996	336	95	0.375783	0.167142	0.208641	702.5565	UNCX	Promoter
chr15	92734189	92734271	83	38	0.464476	0.264964	0.199512	235.1675	FAM174B	Promoter
chr22	39557496	39557807	312	94	0.323181	0.125939	0.197241	688.895	CACNA1I	Distal Intergenic
chr19	58034424	58034618	195	31	0.451697	0.255171	0.196526	182.7757	ZSCAN1	Promoter

chr12	30796214	30796545	332	74	0.236869	0.046857	0.190012	604.5896	LINC00941	Promoter
chr2	25340027	25340154	128	17	0.585193	0.396936	0.188257	76.24683	DNMT3A	Promoter
chr19	50694773	50695172	400	91	0.35097	0.167212	0.183758	517.2599	SHANK1	Intron
chr17	48550385	48550653	269	50	0.780723	0.597517	0.183206	318.8515	HOXB-AS3	Promoter
chr13	23547065	23547231	167	29	0.367943	0.188401	0.179542	167.2475	TNFRSF19	Distal Intergenic
chr16	46427850	46427989	140	22	0.693361	0.518939	0.174422	113.4511	ANKRD26P1	Distal Intergenic
chr1	2.46E+08	2.46E+08	103	13	0.646139	0.473342	0.172797	62.37419	KIF26B	Intron
chr2	9757627	9757725	99	11	0.378756	0.206896	0.17186	53.96079	LOC100996549	Exon
chr10	1.21E+08	1.21E+08	212	28	0.28507	0.114288	0.170782	187.9236	LOC105378519	Intron
chr1	34164815	34165425	611	153	0.247116	0.076451	0.170665	1427.521	CSMD2	Promoter
chr12	1.3E+08	1.3E+08	73	8	0.853761	0.684254	0.169507	36.11068	FZD10-AS1	Distal Intergenic
chr2	1.14E+08	1.14E+08	314	103	0.337738	0.169903	0.167835	764.5855	DDX11L2	Promoter
chr10	1672574	1672756	183	23	0.776396	0.608922	0.167474	121.1247	ADARB2	Intron
chr11	69985640	69985885	246	60	0.242935	0.076393	0.166542	460.1514	ANO1	Promoter
chr21	45405588	45406112	525	179	0.264013	0.098294	0.165719	1004.182	COL18A1	Promoter
chr14	1.02E+08	1.02E+08	449	91	0.243563	0.082401	0.161161	568.3971	PPP2R5C	Intron
chr4	42151345	42152369	1025	283	0.229269	0.069882	0.159387	2239.941	BEND4	Promoter
chr9	1.24E+08	1.24E+08	150	55	0.241063	0.08206	0.159003	361.8002	DENND1A	Promoter
chr2	91685875	91686139	265	52	0.454497	0.297527	0.15697	288.7374	LSP1P4	Exon
chr2	25340414	25340601	188	33	0.239117	0.082344	0.156773	202.7447	DNMT3A	Promoter
chr1	21937458	21937528	71	17	0.270913	0.114508	0.156405	73.09101	HSPG2	Promoter
chr12	1.13E+08	1.13E+08	556	143	0.213724	0.058287	0.155436	1076.825	DTX1	Promoter
chr6	1.67E+08	1.67E+08	91	9	0.730123	0.575087	0.155036	35.73281	TCP10L2	Intron

chr4	5051235	5052080	846	198	0.230597	0.07606	0.154536	1598.494	STK32B	Promoter
chr19	50668428	50668783	356	89	0.308532	0.155129	0.153402	519.1106	SHANK1	Promoter
chr7	1.51E+08	1.51E+08	924	203	0.263368	0.111507	0.151861	1224.358	WDR86	Promoter
chr17	52159346	52159463	118	17	0.267213	0.117974	0.149238	83.85568	CA10	Promoter
chr15	79089887	79090367	481	86	0.217166	0.070421	0.146745	604.5653	RASGRF1	Promoter
chr19	31348656	31349133	478	114	0.221396	0.076247	0.145149	713.2079	TSHZ3-AS1	Promoter
chr1	75615908	75616185	278	72	0.203156	0.058272	0.144884	502.0016	SLC44A5	Distal Intergenic
chr2	2.41E+08	2.41E+08	147	42	0.247618	0.103148	0.144471	204.7586	KIF1A	Promoter
chr19	39265052	39265380	329	67	0.269544	0.125286	0.144259	441.8474	IFNL2	Exon
chr19	50639271	50639397	127	22	0.217315	0.074332	0.142982	123.2171	SYT3	Promoter
chr10	1.33E+08	1.33E+08	376	95	0.285622	0.14275	0.142873	588.8817	NKX6-2	Promoter
chr9	1.37E+08	1.37E+08	260	56	0.391054	0.248886	0.142169	268.7155	LCNL1	Promoter
chr19	50658883	50659223	341	87	0.272484	0.130993	0.141491	542.0834	C19orf81	Promoter
chr19	17328489	17328629	141	33	0.32153	0.180095	0.141435	146.9623	ANO8	3' UTR
chr11	40293096	40293158	63	5	0.257441	0.117063	0.140378	31.31618	LRRC4C	Promoter
chr12	1.13E+08	1.13E+08	149	34	0.309186	0.168907	0.140279	180.3558	RPH3A	Promoter
chr1	57423822	57424446	625	171	0.249189	0.109606	0.139584	1203.075	DAB1	Promoter
chr1	2.31E+08	2.31E+08	152	24	0.277637	0.138055	0.139582	125.7845	TRIM67	Promoter
chr16	58463647	58464056	410	122	0.212818	0.073421	0.139397	796.3722	NDRG4	Promoter
chr1	1.57E+08	1.57E+08	189	30	0.247451	0.108243	0.139209	170.8529	BCAN	Promoter
chr10	77637854	77638255	402	136	0.194749	0.056342	0.138407	985.5173	KCNMA1	Promoter
chr18	46756436	46756765	330	78	0.224761	0.086979	0.137782	518.732	ST8SIA5	Promoter
chr13	1.08E+08	1.08E+08	122	21	0.280765	0.14414	0.136626	106.5921	MYO16	Distal Intergenic
chr6	98824679	98824926	248	45	0.262509	0.128347	0.134162	274.6953	POU3F2	Distal

										Intergenic
chr11	68043720	68043799	80	9	0.842339	0.709574	0.132765	36.98805	TCIRG1	Promoter
chr8	76682021	76682441	421	57	0.256651	0.124417	0.132234	291.9201	ZFHX4	Promoter
chr4	6221994	6222202	209	60	0.283833	0.152146	0.131687	374.7285	JAKMIP1-DT	Promoter
chr7	31052990	31053154	165	27	0.24191	0.110889	0.131021	144.8573	ADCYAP1R1	Promoter
chr4	1.84E+08	1.84E+08	168	44	0.302646	0.171786	0.13086	248.1262	STOX2	Promoter
chr17	20840933	20841138	206	45	0.24545	0.114976	0.130474	233.0273	CCDC144NL	Exon
chr22	43343624	43343943	320	68	0.242161	0.112482	0.129679	411.6842	SCUBE1	Promoter
chr8	11709725	11709854	130	16	0.25359	0.124024	0.129566	77.53641	GATA4	Intron
chr16	51150782	51151237	456	90	0.256718	0.127555	0.129162	524.7594	SALL1	Promoter
chr2	2.32E+08	2.32E+08	827	217	0.190031	0.06103	0.129001	1508.809	ECEL1	Promoter
chr11	70149871	70149949	79	7	0.805722	0.677677	0.128045	29.52599	ANO1	Intron
chr19	8832273	8832811	539	97	0.190736	0.062846	0.12789	543.6526	ZNF558	Promoter
chr17	19978296	19978391	96	16	0.291398	0.163802	0.127595	79.5187	AKAP10	Promoter
chr1	2629227	2629373	147	33	0.243579	0.116093	0.127486	189.4388	MMEL1	Promoter
chr7	98869586	98869899	314	59	0.214024	0.086934	0.12709	343.7496	TMEM130	Promoter
chr10	99529977	99530095	119	26	0.328672	0.202524	0.126148	123.5972	LINC01475	Promoter
chr22	48489799	48490522	724	168	0.193987	0.068695	0.125292	1112.073	FAM19A5	Promoter
chr1	4654561	4654823	263	104	0.313767	0.18871	0.125057	615.6867	AJAP1	Promoter
chr12	1.14E+08	1.14E+08	110	16	0.223185	0.098394	0.124791	73.78764	LOC105369998	Distal Intergenic
chr15	78620580	78621139	560	161	0.197996	0.073315	0.124681	1155.272	CHRNA3	Promoter
chr8	10729970	10730401	432	109	0.181531	0.057389	0.124142	745.4511	SOX7	Promoter
chr15	33310616	33311274	659	158	0.240799	0.118367	0.122432	976.9845	RYR3-DT	Promoter
chr11	40293251	40293314	64	5	0.200353	0.07823	0.122123	24.77055	LRRC4C	Promoter
chrX	23333209	23333381	173	43	0.391215	0.269531	0.121683	196.9223	PTCHD1	Promoter

chr16	705039	705138	100	22	0.277519	0.156099	0.12142	98.91003	FBXL16	Promoter
chr22	44331709	44331785	77	21	0.353014	0.231747	0.121266	85.7899	SHISAL1	Distal Intergenic
chr19	52535853	52536009	157	31	0.312031	0.190967	0.121063	148.5515	ZNF808	Promoter
chr16	28062881	28063359	479	133	0.193231	0.072213	0.121018	879.4091	GSG1L	Promoter
chr4	1.77E+08	1.77E+08	203	48	0.169554	0.049109	0.120445	276.5459	VEGFC	Promoter
chr18	77251544	77251804	261	51	0.246426	0.126872	0.119553	280.2724	GALR1	Promoter
chr3	48662340	48662527	188	25	0.229727	0.110329	0.119398	129.3703	CELSR3	Promoter
chr8	1.43E+08	1.43E+08	139	23	0.263747	0.144491	0.119256	96.96774	LY6H	Promoter
chr10	1.33E+08	1.33E+08	331	78	0.190334	0.071285	0.119049	558.5326	NKX6-2	Promoter
chr12	64824437	64825455	1019	196	0.168323	0.050146	0.118177	1226.203	TBC1D30	Promoter
chr10	80356690	80356905	216	49	0.217026	0.099042	0.117984	248.7593	DYDC1	Promoter
chr13	24988970	24989035	66	6	0.339888	0.222899	0.116989	31.88658	TPTE2P1	Distal Intergenic
chr20	41366389	41366766	378	127	0.198569	0.081996	0.116573	795.8122	EMILIN3	Promoter
chr6	1.28E+08	1.28E+08	319	64	0.164846	0.048552	0.116294	377.5272	SOGA3	Promoter
chr15	88256961	88257341	381	84	0.179985	0.065114	0.114871	466.6567	NTRK3	Promoter
chr8	1.43E+08	1.43E+08	91	16	0.215417	0.100682	0.114735	74.70903	MAFA	Promoter
chr15	88769541	88769663	123	23	0.224133	0.110925	0.113208	111.3911	LOC105370960	Distal Intergenic
chr8	38787798	38787958	161	38	0.288079	0.175185	0.112895	204.5323	TACC1	Promoter
chr17	36402467	36402561	95	21	0.940445	0.827887	0.112558	79.86522	TBC1D3H	Distal Intergenic
chr11	1748836	1749246	411	111	0.198761	0.0866	0.112161	582.0999	IFITM10	Promoter
chr4	84497454	84497594	141	25	0.202205	0.090879	0.111326	132.364	NKX6-1	Promoter
chr17	28371974	28372266	293	74	0.164513	0.054294	0.110219	453.2853	SARM1	Promoter

chr14	97218552	97218837	286	35	0.186966	0.077006	0.10996	177.7166	LINC02304	Exon
chr7	1.58E+08	1.58E+08	567	123	0.23175	0.121795	0.109955	630.1909	PTPRN2	Intron
chr19	37468880	37469139	260	40	0.14763	0.038287	0.109343	244.7864	ZNF570	Promoter
chr15	88604904	88605039	136	17	0.180404	0.071409	0.108995	82.56943	LINC01586	Promoter
chr19	53132613	53132935	323	49	0.179457	0.071446	0.108011	255.5908	ZNF415	Promoter
chr18	75204641	75204816	176	50	0.264748	0.156918	0.10783	264.0497	PTGR3	Promoter
chr2	42047385	42047757	373	98	0.162106	0.05611	0.105996	554.1262	PKDCC	Promoter
chr19	19866785	19866925	141	18	0.144859	0.038866	0.105993	86.8719	ZNF253	Promoter
chr13	26051267	26051724	458	103	0.188324	0.0831	0.105223	645.803	SHISA2	Promoter
chr11	1.12E+08	1.12E+08	721	146	0.181715	0.07657	0.105145	810.1392	LAYN	Promoter
chr7	69599513	69599577	65	17	0.181937	0.076811	0.105126	77.42153	AUTS2	Promoter
chr17	48578036	48578280	245	55	0.154339	0.049543	0.104796	338.0882	HOXB4	Promoter
chr6	1.66E+08	1.66E+08	193	34	0.212042	0.108671	0.103371	180.7581	TBXT	Promoter
chr20	4822500	4823629	1130	214	0.15311	0.050072	0.103037	1232.466	RASSF2	Promoter
chr14	38255798	38255945	148	38	0.231359	0.128599	0.10276	175.8984	CLEC14A	Promoter
chr13	96091018	96091192	175	44	0.182287	0.079871	0.102415	252.1658	HS6ST3	Promoter
chr7	1237512	1237661	150	54	0.196687	0.094486	0.102201	307.7394	UNCX	Downstream
chr3	13282285	13283135	851	261	0.180411	0.078968	0.101442	1455.616	IQSEC1	Promoter
chr7	1.41E+08	1.41E+08	488	102	0.136178	0.035547	0.100631	640.4525	TMEM178B	Promoter
chr15	30196055	30196477	423	87	0.180556	0.08006	0.100496	422.8263	LINC02249	Promoter

Supplementary Table 4. Methylation levels of the DMRs on *DAB1*, *PPP2R5C* and *FAM19A5* genes in 20 pairs of cancer and normal tissues

Tissues	n	*DAB1*[1]	*PPP2R5C*[1]	*FAM19A5*[1]
CRC	20	0.066±0.10	0.055±0.09	0.069±0.08
Healthy controls	20	0.009±0.03	0.003±0.09	0.005±0.01
P-value (T-test)		***0.026***	***0.013***	***0.003***

[1]: Methylation levels are expressed as Mean ± SD

Supplementary Table 5.1. Relationship between methylation of the DMR on *DAB1* gene in cfDNA and clinicopathological characteristics of colorectal cancer

Clinicopathological characteristics	cfDNA		OR（95%CI）	*P*-value
	methylation（%）	Non methylation（%）		
UICC stage				0.575
I- II	24（66.7）	18（60.0）	1	
III-IV	12（33.3）	12（40.0）	1.333（0.487-3.649）	
T stage				0.479
T1-T2	9（23.7）	5（16.7）	1	
T3-T4	29（76.3）	25（83.3）	1.552（0.459-5.241）	
N stage				0.889
N0	26（68.4）	21（70.0）	1	
N1/N2	12（31.6）	9（30.0）	0.929（0.329-2.622）	
M stage				1.000
M0	38（100)	29（96.7)	1	
M1	0（0)	1（3.3)		
Pathological type				0.615
protruded type	4（10.8)	2（7.1)	1	
Ulcerative type	33（89.2)	26（92.9)	1.576（0.267-9.283)	
Differentiation				0.904
Poorly differentiated	6（17.1)	4（13.8)	1	
Intermediate differentiation	24（68.6)	20（69.0)	1.250（0.309-5.056)	
Well differentiated	5（14.3)	5（17.2)	1.500（0.255-8.817)	
Perineural invasion				0.170
Yes	24（66.7)	23（82.1)	1	
No	12（33.3)	5（17.9)	0.435（0.132-1.429)	
Vascular invasion				***0.014***
Yes	11（29.7)	17（60.7)	1	
No	26（70.3)	11（39.3)	0.274（0.097-0.771)	

Supplementary Table 5.2. Relationship between methylation of the DMR on *PPP2R5C* gene in cfDNA and clinicopathological characteristics of colorectal cancer

Clinicopathological characteristics	cfDNA		OR（95%CI）	*P*-value
	methylation（%）	Non methylation（%）		
UICC stage				0.885
I- II	15（62.5)	27（64.3)	1	
III-IV	9（37.5)	15（35.7)	1.080（0.382-3.055)	
T stage				0.690
T1-T2	6（23.1)	8（19.0)	1	
T3-T4	20（76.9)	34（81.0)	0.784（0.238-2.588)	
N stage				0.987
N0	18（69.2)	29（69.0)	1	
N1/N2	8（30.8)	13（31.0)	0.991（0.344-2.859)	
M stage				1.000
M0	25（96.2)	42（100)	1	
M1	1（3.8)	0（0)		
Pathological type				0.731
protruded type	3（12.5)	4（9.8)	1	
Ulcerative type	21（87.5)	37（90.2)	0.757（0.154-3.710)	
Differentiation				0.729
Poorly differentiated	4（16.0)	6（15.4)	1	
Intermediate differentiation	16（64.0)	28（71.8)	0.857（0.210-3.498)	
Well differentiated	5（20.0)	5（12.8)	1.500（0.255-8.817)	
Perineural invasion				0.058
Yes	21（87.5)	26（65.0)	1	
No	3（12.5)	14（35.0)	0.265（0.067-1.047)	
Vascular invasion				0.488
Yes	9（37.5)	19（46.3)	1	
No	15（62.5)	22（53.7)	1.439（0.514-4.030)	

Supplementary Table 5.3. Relationship between methylation of the DMR on *FAM19A5* gene in cfDNA and clinicopathological characteristics of colorectal cancer

Clinicopathological characteristics	cfDNA		OR（95%CI）	*P*-value
	methylation（%）	Non methylation（%）		
UICC stage				0.842
I- II	13（61.9）	29（64.4）	1	
III-IV	8（38.1）	16（35.6）	1.115（0.382-3.257）	
T stage				0.349
T1-T2	6（27.3）	8（17.4）	1	
T3-T4	16（72.7）	38（82.6）	0.561（0.168-1.881）	
N stage				0.908
N0	15（68.2）	32（69.6）	1	
N1/N2	7（31.8）	14（30.4）	1.067（0.357-3.189）	
M stage				1.000
M0	21（95.5）	46（100）	1	
M1	1（4.5）	0（0）		
Pathological type				0.302
protruded type	1（4.8）	6（13.6）	1	
Ulcerative type	20（95.2）	38（86.4）	3.158（0.335-28.080	
Differentiation				0.999
Poorly differentiated	17（81.0）	27（62.8）		
Intermediate differentiation	0（0）	10（23.3）	1	
Well differentiated	4（19.0）	6（14.0）		
Perineural invasion				0.075
Yes	17（89.5）	30（66.6）	1	
No	2（10.5）	15（33.3）	0.235（0.048-1.155）	
Vascular invasion				0.454
Yes	10（50.0）	18（40.0）	1	
No	10（50.0）	27（60.0）	0.667（0.231-1.925）	
